# Bias in pharmacoepidemiologic studies using secondary health care databases: a scoping review

**DOI:** 10.1186/s12874-019-0695-y

**Published:** 2019-03-11

**Authors:** Guillermo Prada-Ramallal, Bahi Takkouche, Adolfo Figueiras

**Affiliations:** 1 Department of Preventive Medicine and Public Health, University of Santiago de Compostela, c/ San Francisco s/n, 15786 Santiago de Compostela, A Coruña Spain; 2 Health Research Institute of Santiago de Compostela (Instituto de Investigación Sanitaria de Santiago de Compostela - IDIS), Clinical University Hospital of Santiago de Compostela, 15706 Santiago de Compostela, Spain; 3 Consortium for Biomedical Research in Epidemiology & Public Health (CIBER en Epidemiología y Salud Pública – CIBERESP), Santiago de Compostela, Spain

**Keywords:** Pharmacoepidemiology, Observational studies, Bias, Confounding factors, Medical records, Electronic health records, Administrative claims, Medical record linkage

## Abstract

**Background:**

The availability of clinical and therapeutic data drawn from medical records and administrative databases has entailed new opportunities for clinical and epidemiologic research. However, these databases present inherent limitations which may render them prone to new biases. We aimed to conduct a structured review of biases specific to observational clinical studies based on secondary databases, and to propose strategies for the mitigation of those biases.

**Methods:**

Scoping review of the scientific literature published during the period 2000–2018 through an automated search of MEDLINE, EMBASE and Web of Science, supplemented with manually cross-checking of reference lists. We included opinion essays, methodological reviews, analyses or simulation studies, as well as letters to the editor or retractions, the principal objective of which was to highlight the existence of some type of bias in pharmacoepidemiologic studies using secondary databases.

**Results:**

A total of 117 articles were included. An increasing trend in the number of publications concerning the potential limitations of secondary databases was observed over time and across medical research disciplines. Confounding was the most reported category of bias (63.2% of articles), followed by selection and measurement biases (47.0% and 46.2% respectively). Confounding by indication (32.5%), unmeasured/residual confounding (28.2%), outcome misclassification (28.2%) and “immortal time” bias (25.6%) were the subcategories most frequently mentioned.

**Conclusions:**

Suboptimal use of secondary databases in pharmacoepidemiologic studies has introduced biases in the studies, which may have led to erroneous conclusions. Methods to mitigate biases are available and must be considered in the design, analysis and interpretation phases of studies using these data sources.

**Electronic supplementary material:**

The online version of this article (10.1186/s12874-019-0695-y) contains supplementary material, which is available to authorized users.

## Background

In recent decades, with advances of computer technology and the exponential growth in the quantity of data available, new opportunities for research in many fields have emerged. One of these fields is the health sector, due to the availability of clinical and therapeutic data drawn from medical records and administrative databases used for billing and other fiscal functions related to the provision of patient care (i.e. secondary databases) [1].

This availability of data has increased the interest of pharmacoepidemiologists in using secondary databases as sources of data for research. Contributing to this is the perception that clinical trials are not always useful for evaluation of therapies in real-world practice, particularly those providing limited safety data. However, swift and easy access to this information may be deceptively simple [2]. Indeed, the utilization of secondary databases entail not only the limitations specific to observational epidemiologic research but those inherent to these specific types of sources [3], as well as the social and ethical challenges related to data privacy and security [4, 5].

Consequently, many researchers recommend caution and warn against the high risk of introducing biases when using these databases [6–9]. The aim of this study was thus to review the literature of the last two decades in which the authors highlight the existence of some type of bias in observational clinical studies based on secondary data sources, in order to identify the most common biases and explore the perception of this issue in the pharmacoepidemiologic field over time and across medical research disciplines. We then propose possible strategies to control the biases identified in the review.

## Methods

We carried out a scoping review, which is a methodological strategy that enables the results of an exploratory research to be summarized. In this type of review, unlike other systematic reviews, the application of quality filters is not an initial priority [10]. We performed and reported our study based on the methodological guidance for the conduct of a scoping review from the Joanna Briggs Institute [11] and the PRISMA (Preferred Reporting Items for Systematic reviews and Meta-Analyses) Extension guideline for Scoping Reviews [12]. The protocol for this scoping review is available on request from the corresponding author.

### Data-sources and search strategy

An automated search of bibliographic databases was performed, with an initial search in MEDLINE, subsequently supplemented by EMBASE and Web of Science. To avoid duplicated results, in EMBASE and Web of Science we used the option that enables journals indexed in MEDLINE to be excluded. The same free-text search strategy was applied in the 3 databases: (clinical–data* OR health–data* OR medical–data* OR prescription–data* OR administrative–data* OR epidemiologic–data* OR health–claim* OR administrative–claim* OR insurance–claim* OR claims–data* OR health–record* OR medical–record*) AND (confounding OR bias* OR missing–data OR misclassification) AND (observational OR epidemiolog* OR pharmacovigilance OR challenge*) AND drug, from January 1, 2000 to January 1, 2018. All types of research design were considered. Adding restrictive MeSH (Medical Subject Headings) terms according to type of publication was not deemed suitable, since this was found to lead to an excessive reduction in search sensitivity.

Once the references were identified, the titles and the abstracts, when available, were used as a preliminary screening filter, and if deemed potentially relevant, full text articles were retrieved. Other relevant references were identified by manually cross-checking reference lists of selected articles and using the “related articles” option. This full screening was performed by two reviewers (GP-R, AF). Discrepancies were discussed between the two reviewers to achieve consensus. In case of a possible disagreement, a third author (BT) was designated.

### Article selection and data abstraction

We included in the review opinion essays, methodological reviews, analyses/reanalyses and simulation studies, as well as letters to the editor or retractions, the principal objective of which, described in their abstracts, was to highlight the existence of some type of bias in pharmacoepidemiologic studies that used secondary health care databases.

In order to reduce the number of identified references and thus simplify the display of the results, the following exclusion criteria were considered that classified dismissed references into subgroups: (1) its principal objective was to describe, compare, evaluate, validate or develop a bias-control strategy for a known bias or limitation (e.g. analytical method, study design, algorithm, framework); (2) it estimated a measurement (e.g. association treatment-effect) or identified risk factors for a disease, with the existence of bias being mentioned as a limitation of the study, regardless of whether or not strategies for its control were used; (3) it had characteristics different from those indicated above (e.g. studies with different objectives, not based on secondary databases, with no drug involved, no bias mentioned) or it was a conference paper with no abstract/full-text available.

A data charting form was jointly developed by two reviewers (GP-R and AF) to determine which variables had to be extracted. One person (GP-R) extracted the information from the articles (i.e. first author, publication date, category under which the journal was indexed −if the journal was indexed under more than one category, the category under which it was best ranked was considered−, type of article, type of bias(es) mentioned) and when further clarification was needed, articles were checked and validated by additional reviewers as a form of quality control (AF and BT). The three reviewers discussed the results and continuously updated the data charting form.

The synthesis included both quantitative analysis (i.e. publication trend of identified/included articles and frequency analysis of the biases mentioned) and qualitative analysis (i.e. content analysis) of the components of the research purpose.

## Results

**Fig. 1:**
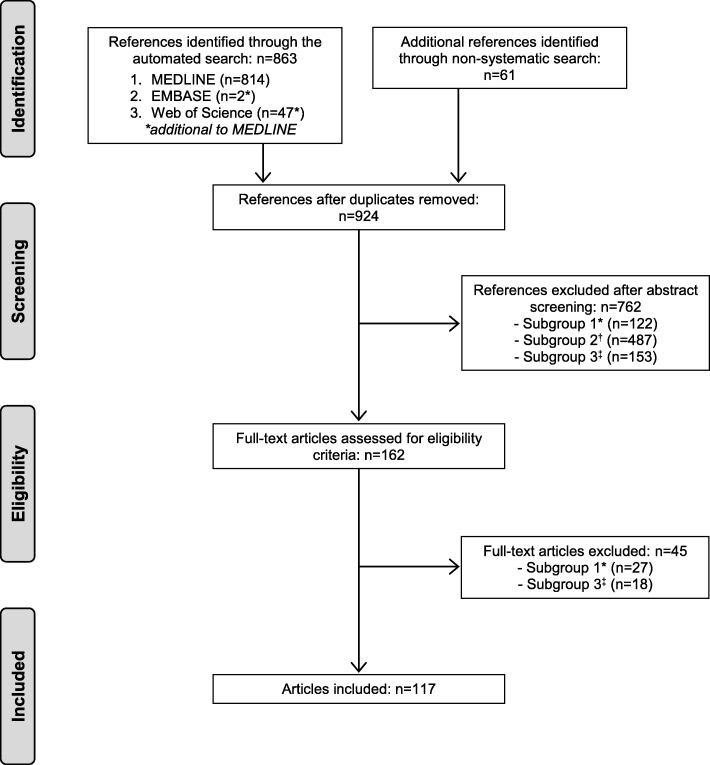
Flow chart of the article selection process. ^*^ Subgroup 1: Its principal objective was to describe, compare, evaluate, validate or develop a bias-control strategy for a known bias or limitation. ^†^ Subgroup 2: Estimated a measurement or identified risk factors for a disease, with the existence of bias being mentioned as a limitation of the study, regardless of whether or not strategies for its control were used. ^‡^ Subgroup 3: Had characteristics different from those indicated above or was a conference paper with no abstract/full-text available

Figure 1 shows the article selection process. A total of 117 articles were included. The automated search resulted in the identification of 863 non-duplicated references, which were reduced to 56 after application of the exclusion criteria. The manual selection process incorporated a further 61 references.

### Publication trend

**Fig. 2:**
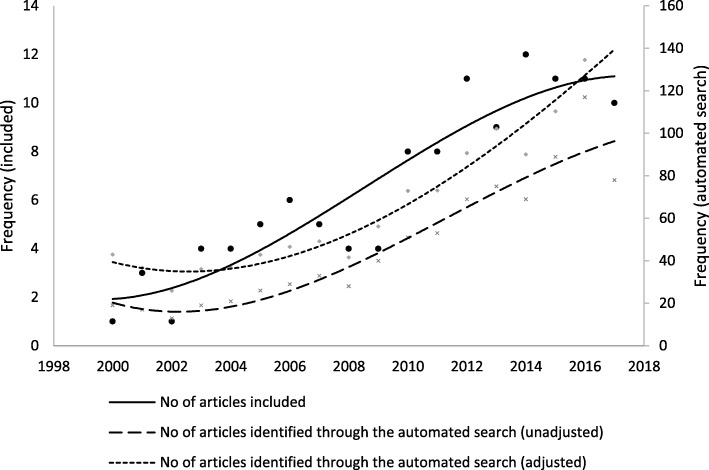
Publication timeline of the 117 articles included in the review (left Y axis) and the 863 references identified through the automated search (right Y axis) unadjusted and adjusted by the number of indexed citations added to MEDLINE

Figure 2 shows a polynomial smoothing of the frequency with which the articles included in the review were published since 2000. An increasing trend is observed, so that nearly half (45.3%, 53/117) of the articles were published during the last 5 full years of this review. There is a similar trend in the timeline of references identified through the automated search when adjusted by the number of indexed citations added to MEDLINE during each year [13], which suggests that the restriction criteria considered did not introduce any selection bias. A slight decrease in 2017 may be due to inherent characteristics of the indexing process in the bibliographic databases, or to the fact that the most recent references have had less time to be cited, and consequently are less likely to be identified by the cross-reference manual search.

**Fig. 3:**
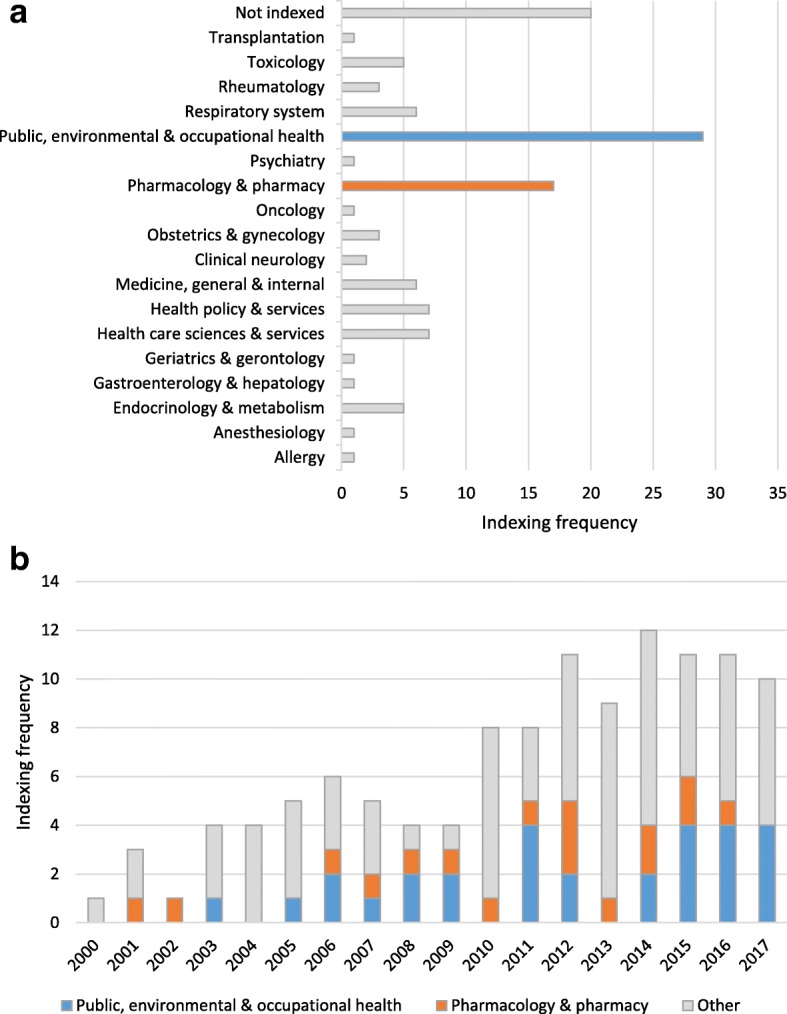
**a** Distribution of included articles across medical disciplines. **b** Timeline of included articles by most prevalent indexed disciplines

There seems to be a wide variety of disciplines interested in articles about the potential limitations of secondary databases (see Fig. 3a). Overall, the most frequently used categories of medical journals were “Public, environmental & occupational health” (24.8%, 29/117 articles included) and “Pharmacology & pharmacy” (14.5%, 17/117). In general, the same publication trend over time is observed when stratifying by discipline (see Fig. 3b).

### Major biases mentioned in the articles included in the review

Table 1 lists the articles that mentioned the categories or subcategories of the biases most usually described in observational studies of pharmacoepidemiologic databases. Confounding bias as such, or in any of its diverse forms of presentation, was the most frequently mentioned category of bias (63.2%, 74/117 articles included), while confounding by indication was the most frequent subcategory (32.5%, 38/117) followed by unmeasured/residual confounding (28.2%, 33/117). Mention was also made of time-dependent confounding and over-adjustment due to inappropriate choice of variables in the statistical model (bias from misspecification of control variables).

**Table 1:** Articles that mention the most usual biases described in observational studies of pharmacoepidemiologic databases

Category/Subcategory	Description of the bias	References (*n* = 117)	Percentage (%)
Confounding	The measure of association between treatment and outcome is distorted by the effect of one or more variables, which are also risk factors for the outcome of interest	[1–3, 6, 14–16, 18, 22, 40, 41, 57, 58, 62, 80–139]	63.2
Confounding by indication^a^	The clinical condition that determined the prescription of the treatment is associated with the effect, acting as a confounding factor (e.g. a worse disease status at baseline: confounding by disease severity)	[3, 6, 18, 22, 40, 41, 57, 80, 82, 84, 86, 87, 89, 90, 92, 96, 97, 99, 100, 104, 106, 107, 110, 111, 113, 114, 116, 118, 120, 122, 126, 128–131, 133, 134, 138]	32.5
Time-dependent confounding	A variable that can vary with time acts as a confounding factor between the current exposure and outcome, and as an intermediary between prior and current exposure	[40, 41, 57, 58, 81, 92, 104]	6.0
Unmeasured/residual confounding	There is not enough information about all the relevant confounding factors known, unknown or difficult to measure (e.g. frailty). If confounding cannot be completely controlled for, the residual confounding effect of some factors remains in the final effect that is observed	[1–3, 6, 14, 15, 18, 58, 62, 80–83, 86, 89, 91–93, 96, 101, 103, 108, 110, 113, 116, 119, 125, 127, 130, 132, 134, 136, 139]	28.2
Healthy user/adherer effect	Access to health care resources is associated with a higher level of education and health-seeking behavior. Furthermore, patients who comply with the treatment during prolonged periods of time tend to be healthier	[2, 18, 91, 96, 125, 127]	5.1
Selection bias	The study sample population is not representative of the target population to which the results will be extrapolated	[2, 16, 18, 22, 40, 41, 54, 57, 58, 63, 81, 83, 84, 87, 88, 90, 91, 93–95, 99, 101–103, 105, 107–109, 111–113, 115–119, 121, 122, 124, 125, 135–137, 140–151]	47.0
Protopathic bias	The treatment is associated with subclinical disease stages (an early manifestation of the still undiagnosed condition under study gives rise to prescription of the treatment)	[40, 41, 81, 109]	3.4
Losses to follow-up (informative censoring)	The mechanism that triggers discontinuity of the treatment is associated with the risk of observing the outcome of interest	[40, 41, 116]	2.6
Depletion of susceptibles (prevalent user bias)	The inclusion of prevalent instead of incident users entails insufficient verification of the adverse effects that occur at the beginning of treatment (those susceptible to the adverse effect have interrupted the treatment)	[2, 40, 41, 57, 83, 90, 99, 107, 111, 116, 118, 148]	10.3
Missing data	In multivariate analyses, such as regression models, observations that lack one or more of the values of a variable included in the model tend to be eliminated	[58, 63, 87, 93, 94, 108, 112, 116, 119, 125, 135–137, 140, 141, 143–147, 151]	17.9
Measurement bias	Data on true exposures, outcomes and other variables are recorded in the form of indicators (observed measures) that do not accurately reflect reality	[2, 3, 6, 7, 16, 40, 41, 54, 55, 58, 87, 88, 91, 93, 94, 96, 101, 105, 108, 110, 112, 114, 115, 117, 119, 121, 124, 125, 130, 135–138, 140, 141, 143, 144, 146, 147, 149, 151–164]	46.2
Misclassification bias	The association between treatment and outcome is distorted by systematic errors, due to the way in which the variables of interest are measured in comparison groups	[2, 3, 6, 7, 16, 40, 41, 54, 55, 58, 87, 88, 91, 93, 94, 96, 101, 105, 108, 110, 112, 114, 115, 119, 121, 125, 130, 135–138, 140, 141, 143, 144, 146, 147, 149, 152–164]	43.6
Misclassification of exposure	The measure of exposure of a given treatment is not an exact reflection of its real use (e.g. flawed measurement, non-compliance with treatment, inappropriate use of time windows)	[2, 3, 16, 40, 41, 54, 55, 58, 87, 91, 93, 94, 96, 101, 110, 119, 121, 130, 138, 140, 146, 147, 152, 154, 156, 158, 159, 164]	23.9
Misclassification of outcome	Error in the diagnosis (e.g. clinical ambiguity, non-uniform coding)	[2, 3, 6, 7, 16, 40, 41, 54, 58, 87, 91, 93, 94, 96, 101, 110, 112, 114, 121, 125, 135–137, 141, 143, 149, 153, 155, 157, 160–163]	28.2
Time-related bias	Follow-up time and exposure status are inadequately taken into account in the study-design or analysis stages	[2, 7, 40, 41, 57, 68–75, 77, 83, 86, 87, 90, 99, 101, 105–107, 111, 114, 118, 128, 129, 133, 142, 165–170]	30.8
Immortal time bias	A period of time (immortal) during which the study event cannot occur is included in the follow-up or is excluded from analysis due to an incorrect definition of the start of follow-up	[2, 7, 40, 41, 57, 68–75, 77, 83, 86, 87, 90, 99, 101, 106, 107, 111, 114, 118, 128, 129, 133, 166, 167]	25.6
Immeasurable time bias	A period of time (immeasurable) during follow-up is ignored and thus misclassified as unexposed period, since outpatient prescriptions that define exposure cannot occur (e.g. serious chronic diseases that require extensive use of medications and multiple hospitalizations)	[142, 165, 168, 170]	3.4
Time-window bias	The use of time-windows of different lengths between cases and controls to define time-dependent exposures prevents subjects from having the same opportunity time to receive prescriptions	[90, 106, 169]	2.6
Time-lag bias	Comparisons are conducted of treatments given at different stages of the disease, which inherently introduces bias related to disease duration and progression	[106]	0.9

^a^Sometimes also referred to as channeling bias

Similarly, some type of selection and measurement bias was mentioned in 47.0% (55/117) and 46.2% (54/117) of the articles included, respectively. Bias due to missing data and prevalent user bias were the most frequently reported selection biases (38.2%, 21/55 and 21.8%, 12/55, respectively); in addition, other forms of bias were also described, such as protopathic bias, informative censoring, competing risks, and differential health care access bias. Exposure or outcome misclassification were the most usual causes of measurement bias (51.9%, 28/54 and 61.1%, 33/54 respectively). Temporal ambiguity and misclassification of confounders were likewise cited.

**Fig. 4:**
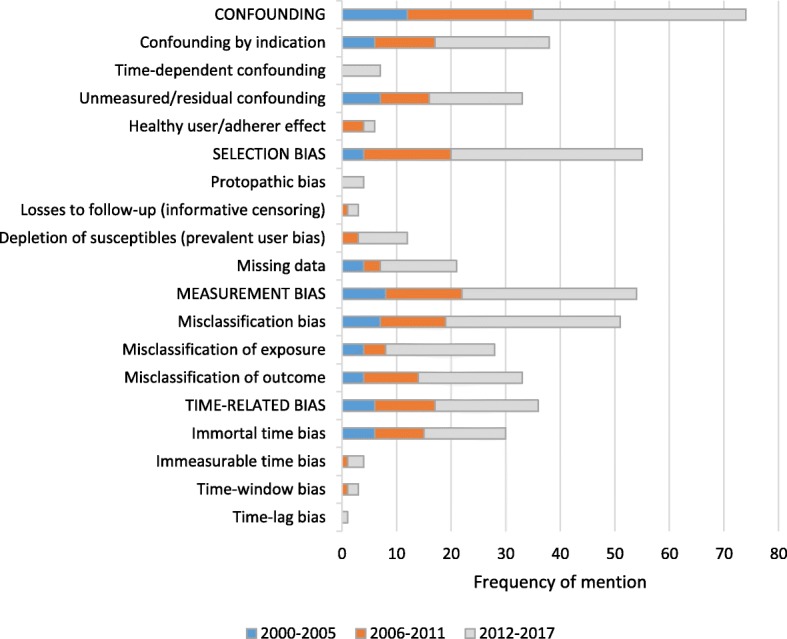
Frequency of the biases mentioned in the included articles stratified by time periods

Although they can strictly be considered a subset of the larger 3 categories (i.e. confounding, selection or measurement bias), last to be examined was the category of time-related biases, such as the “immortal time” bias, which proved to be the single most reported bias (25.6%, 30/117) after confounding by indication, unmeasured/residual confounding and outcome misclassification (28.2%, 33/117). Immeasurable time bias, time-window bias and time-lag bias were also described. Figure 4 shows the frequency for each bias mentioned in the articles included, as well as the overarching categories, stratified by 6-year time periods.

Additional file 1: Table S1 contains the data extracted from the included articles in descending order of publication date by the research field category under which the journal was indexed. The articles were also classified according to type of content, including, in each case, the categories or subcategories of bias mentioned.

## Discussion

This is the first known structured review that explores potential biases in observational studies of pharmacoepidemiologic databases. The results of this review suggest that there is growing concern in the scientific literature about identifying, describing and controlling such biases. This should not be overlooked, since observational epidemiologic database studies currently afford an excellent opportunity for medical research. The results of these studies are to be valid and applicable to decision-making about safety and effectiveness. It is then of paramount importance that proper account be taken of these biases to ensure that they are correctly controlled for.

Confounding bias as such, or in any of its diverse forms of presentation, is mentioned in almost two-thirds of the articles included in the scoping review (see Table 1 for references). Adequate control of confounding poses a challenge in studies that use health care databases, since these were not designed for undertaking epidemiologic studies. The absence or poor quality of data on potential confounding factors in secondary databases (e.g. over-the-counter drugs, frailty of the subject, smoking habit) is a frequent phenomenon [14–17], which renders it difficult or even impossible to adjust for such factors, in order to control for confounding [18].

If data on confounding variables has been collected, the reviewed articles propose different control methods: (1) in the design stage, through the application of restriction criteria, matching methods, or implementation of a new-user design (see below, *depletion of susceptibles*); and (2) in the analysis stage, through stratification of patients across treatment groups according to relevant factors, or multivariate regression techniques, by including these confounding factors as independent variables in regression models. In cases in which the number of variables is very high, adjusting for the disease risk score [19] or the propensity score to receive treatment may be of interest [20, 21].

Among the studies dealing with the issue of confounding in pharmacoepidemiology, the most commonly described type of confounding is *confounding by indication for treatment* (the treatment decision is associated with an indication, which is in turn a risk factor for the disease), which is mentioned in one-third of the articles reviewed (see Table 1). Confounding by indication, often also referred to as channeling bias, is closely related to selection bias [22]. Some useful analytical control methods proposed include separating the effects of a drug taken at different times [23], sensitivity analysis for unmeasured confounding factors (see below), and the use of instrumental variables [24]. Furthermore, according to the literature reviewed, there seems to be a general agreement that conventional methods for control of confounding factors are inadequate in controlling *time-dependent confounding* (mentioned in 6.0% of the articles reviewed, see Table 1). G–estimation [25] and marginal structural models [26] are alternative methods for achieving such control.

More than a quarter of the articles included in the scoping review consider the absence of quality data to control for potential confounding variables as an important limitation of observational pharmacoepidemiologic studies using secondary databases (see Table 1). Therefore, the proposed strategies for the control of *unmeasured variables* include the performance of sensitivity analyses and use of information external to the database [27–29]. Instrumental variable techniques, proxy measures and propensity scores, excluding from the analysis treated and untreated subjects having extreme values, have also been used [30]. In the design stage, case-crossover study designs, where each study participant receives all treatments that are being investigated but at different times [31], and restriction to an active comparison group can be useful. The active comparator design emulates the design of a head to head randomized controlled trial. Instead of using a non-user group, the drug of interest is compared with another drug commonly used for the same indication. By ensuring that treatment groups have similar characteristics, this design potentially helps to mitigate both measured and unmeasured confounding [32]. At all events, with the exception of crossover designs, where the order in which a study participant receives the treatments is randomized, control for unmeasured variables will never be optimal or, at best, one could never be sure that it would be so. But even in this case, the crossover design may still be affected by time-dependent confounding.

In this context, Hernán has proposed a new approach based on the use of observational data from a large health care database to emulate a hypothetical randomized trial (the target trial) [33]. Although the emulated target trial helps avoid common methodologic pitfalls, the appropriate adjustment for time-dependent confounders remains critical [34].

In contrast to clinical trials, an advantage of observational pharmacoepidemiologic studies in which the study populations are constructed on the basis of large health care databases is the inclusion of *frail patients*. However, some authors have argued that due to the fact that frailty is difficult to measure and a strong risk factor for unfavorable outcomes, it will lead to unmeasured and residual confounding, and possibly to paradoxical results [35, 36]. Frailty is an example of an unmeasured confounding variable [14, 15].

About 5% of the reviewed articles deal with the *healthy user effect* (see Table 1), which consists of a type of confounding generated because patients with healthier behaviors generally demand medical attention more frequently for preventive treatments or asymptomatic chronic diseases. These patients are also more likely to be better adherers. Accordingly, part of the apparent efficacy/safety of the treatment will be due, not to the treatment per se, but rather to the healthier behaviors that are associated with those taking it [18, 37]. In observational studies of pharmacoepidemiologic databases, these types of behavior are seldom measured, thus making it very difficult to control for their effect [38].

Almost half of the articles included in the scoping review mention some type of selection bias. Within this category, it is worth highlighting the *protopathic bias*. Although this bias is not widely mentioned in our review (3.4%, see Table 1), possibly because it is unusual for the treatment to be associated with subclinical states and/or early symptoms of the disease, the impact of this bias may be important. However, controlling protopathic bias is not easy since it is not a confounding bias, and adjustment techniques are thus useless. In this case, we must resort to restriction of the exposure group to patients with indications that are unrelated to the initial states of the disease under study. Another option for controlling protopathic bias is to use the concept of lag–time to define the etiologic window in which the exposure to the drug is assessed [39].

Consumption of medicines under real conditions is subject to important variations (e.g. variation in the dose, treatment interruptions, dropouts), especially in the management of chronic diseases. This variability may be due to changes in the disease (increasing or decreasing severity) or in the effect of the drug (adverse events or interactions). The traditional approach through an “as-treated” analysis, in which one censors subjects who interrupt their treatment during follow-up, may introduce bias since censored subjects (losses to follow-up) are systematically at higher or lower risk of developing the outcome [40, 41]. In practice, this *informative censoring* (mentioned in only 2.6% of the articles reviewed, see Table 1) leads to a selection bias. For example, if the clinical effects expected are not met then the treatment is suspended or modified. The bias consists in selecting for the analysis data of patients for whom the treatment produces the expected outcome [42]. This bias may be identified through sensitivity analyses. In this regard, the use of databases represents an important advantage as information on the outcome may be available even when the treatment was suspended. To control the bias introduced by an exposure to the drug that varies with time, it could prove useful to consider that exposure as a time-dependent variable in an appropriate multivariate regression model. Procedures based on the inverse probability censoring weighting have also been proposed [43].

Judging by the number of articles that mention it (10.3%), greater importance has been given to another type of selection bias known as *depletion of susceptibles*, which is caused by the inclusion in the study of both prevalent and incident treatment users (see Table 1). Prevalent users (“survivors” from the first treatment period) may not have the same risk of an adverse event as incident (new) users, i.e., those who tolerate the medication continue using it and those who do not tolerate the medication (susceptible to the adverse event) have stopped using it. This bias can be prevented in the design stage of the study by limiting the follow-up to new users [44]. The new-user design allows potential confounding factors to be measured just before the start of follow-up. This way, these confounding factors will not be affected by the treatment. Adjustment for differences between treatment groups will then use the baseline values of the confounders [45].

Apart from ensuring an appropriate adjustment for confounding, the new-user design potentially reduces immortal time bias (see below) when combined with the active comparator design by implementing similar definitions of the index date across comparison groups [32]. The new user design combined with the active comparator design can also reduce confounding by indication and other unmeasured patient characteristics (e.g. frailty, healthy user) at the design stage [46].

As our results suggest, one of the major challenges in the analysis of observational data is the *missing data* issue [47], which is mentioned in almost one of every five articles included in the scoping review (see Table 1). If the probability of missing an observation is independent of both observed and missing data, complete cases are assumed to be a random sample of the full dataset (i.e. missing completely at random [48]). In this case, dropping cases with missing data may give unbiased estimates. However, in the multivariate analysis, observations (or subjects) are eliminated whenever where data of a variable included in the model are missing. As a consequence, observations with missing values may lead to a substantial attrition of the sample size. If this lack of information is associated with an important characteristic (e.g. severity, frailty), an effect equivalent to selection bias is produced.

Sometimes, it is assumed that the probability of missing an observation may be predicted by variables that are measured previously, but which are not further dependent on unmeasured variables (i.e. missing at random [48]). That is, the probability of dropout will depend on observed values. Although standard analysis of the available cases is potentially biased in this case, methods that can provide valid analysis are available, but these require additional appropriate statistical modeling.

In both circumstances described above, likelihood-based methods (e.g. mixed models), in which missing data can be estimated using the conditional distribution of the other variables, can be useful for controlling bias [49]. There are alternative techniques, such as multiple imputation, that preserve the natural variability of the data [50] and incorporate the uncertainty due to missing data [51], with which similar results are obtained. Inverse probability weighting (where complete cases are weighted by the inverse of their probability of being a complete case) is also a commonly used method to reduce this bias. While multiple imputation requires a model for the distribution of missing data given the observed data, the inverse probability weighting requires a model for the probability that an individual is a complete case [52]. In any case, it is important that all covariates on which missingness depends be included in the model.

On the contrary, if the fact that an observation is missing is predicted by unmeasured variables, such as the outcome of interest (i.e. missing not at random, sometimes called “non-ignorable non-response” or “informative missingness”), then no statistical approach can give unbiased estimates. When missingness cannot be empirically modelled, the recommended approach is to conduct sensitivity analyses to determine the extent of missingness [53].

After confounding by indication and unmeasured/residual confounding, our results show that the bias most frequently described in studies using secondary health care databases is that due to systematic misclassification errors which distort the association between treatment and outcome. *Exposure or outcome misclassification*, which is mentioned in almost half of the articles included in the scoping review (see Table 1), can give rise to measurement biases and heterogeneity [17, 54, 55]. To prevent this, a validation study of these variables should first be conducted, followed by the performance of a sensitivity analysis or application of regression techniques [56]. Medical records are normally considered the *gold standard* or reference for intermediate and final outcome variables but display limitations in the recording of all medications taken by patients [57]. While dispensing records are more detailed in measurement of exposure (though they do not record the over-the-counter or out-of-pocket consumption at an individual level), they nonetheless lack outcome variables [1, 3, 58, 59]. It is therefore important to link both types of data sources [60, 61] and consider, when necessary, the use of additional data collected expressly for research purposes [15, 62, 63], to avoid errors that may generate misleading conclusions [64, 65].

The last category of bias identified was that related to time. However, it must be taken into account that the mechanism that underlies the generation of a time-related bias may be closely related to the other larger categories described (i.e. confounding, selection or measurement bias). By far, the most frequently described time-related bias is the *immortal time bias*, which is mentioned in one of every four articles reviewed (see Table 1). Immortal time bias (where the follow-up includes a time period during which the study event cannot occur or is excluded from the analysis due to an incorrect definition of the start of follow-up) resurged with a number of observational studies that reported surprisingly beneficial effects of drugs [66, 67] and is increasingly being described in cohort studies of pharmacoepidemiologic databases [68–70]. Suissa warns about the risk of reporting absurd conclusions, if inappropriate data-analysis methods are used [69–75]. To prevent this, the entire follow-up time, including that preceding the start of exposure, must be considered, and exposure during immortal time must be correctly classified [76]. By applying a Cox model with time-dependent exposures, more reliable estimates can be obtained [69, 77, 78].

### Limitations

This scoping review presents the limitations inherent to this type of study design. In contrast to classical systematic reviews, the aim of which is to provide answers to a clearly defined research question, the scoping studies are less likely to seek very specific research questions nor, consequently, to assess the quality of included studies [79]. In this sense, a potential reviewer’s bias in the assessment of the restriction criteria cannot be ruled out since they are not based on a measurable quality of the identified references. However, we do not believe that this may hinder the purpose and the conclusions of the review.

Due to the exploratory nature of this review, its purpose was not to obtain all available evidence on a specific topic, but rather that from a subset of the literature on a broad topic (bias in observational pharmacoepidemiologic studies using secondary data sources), where many different study designs might be applicable (opinion essays, methodological reviews, analyses, letters to the editor or retractions). Although a wide-search strategy was employed, some relevant studies may have been missed. Therefore, the existence of some selection bias cannot be ruled out. Furthermore, the search strategy itself, intentionally designed to identify articles that highlight the limitations of secondary databases, does not allow an unbiased comparison with the articles that may show the advantage of secondary databases.

Given the above limitations, and the fact that information on bias was extracted based on the description provided by the original authors, another limitation would be related to the quantification of each type of bias. This should be interpreted as an approximate measure of the impact of the bias on the published literature (i.e. what is prominently talked about), but not as an estimate of the probability of occurrence (or detection) of the bias in the population of pharmacoepidemiologic studies that use secondary databases, since it may be influenced by the ease of describing that specific bias or by the interest that the bias may have raised in the studies of the most prolific authors in the field (e.g. immortal time bias). It is therefore possible that a certain degree of misclassification of some biases exists.

## Conclusions

The emergence of health care databases has caused dramatic changes in pharmacoepidemiology. Due to routine, automated capture of data on drug prescription and dispensing that are used for administration purposes, together with the implementation of electronic medical records, secondary databases have generated enormous possibilities and expectations about their potential. This happens, moreover, at a time when it is recognized that clinical trials cannot answer questions about the effectiveness and safety of treatments in clinical practice.

Superficially, secondary databases afford the possibility of performing studies rapidly, at low cost, with enormous sample sizes, objective data and long-term follow-up. Even so, their limitations should not be ignored. This review provides a complete overview of the potential biases inherent to this type of data sources, including the weighting of their impact on the literature of the last two decades. Confounding by indication, unmeasured/residual confounding, outcome misclassification and immortal time bias are the most important biases. Although this should not be interpreted as an estimate of the risk of those biases, it may indicate which situations have raised greater interest among researchers so far and therefore should be especially considered in future studies using secondary databases to prevent their occurrence.

Appropriate methodological designs and application of statistical analysis techniques must be considered to control such situations. These strategies, summarized in Table 2, are also discussed in this review. In general, before initiating a research using secondary databases, researchers should assess in detail the sources of data available, focusing on the purpose for which they were created, and so become aware of their potential for bias. Medical records linkage with administrative databases can be useful to minimize the risk of bias, as well as the supplement or validation of secondary data with primary data (i.e. collected from ad hoc methods) when the completeness or quality of original data is questionable.

**Table 2:** Main bias-control strategies in observational studies of pharmacoepidemiologic databases

Category	Control strategies
Confounding	
Measured confounding	- Multivariate analysis - Restriction* - Stratification - Matching - New-user design - Propensity score - Large-scale, simple randomized trials - Meta-analysis of clinical trials * *Confounding by indication:* Restricting the untreated group to a population with the same indication, or limiting participation to patients without a risk factor for the effect that could have determined the treatment
Time-dependent confounding	- G–estimation - Marginal structural models
Unmeasured confounding	- Crossover design - Asymmetric exclusion of patients with extreme propensity-score values - Instrumental variables - Proxy measures - Restriction (active comparison group) - Sensitivity analysis - Validation study + external adjustment
Selection bias	
Protopathic bias	- Restriction (e.g. restricting the untreated group to a population with the same indication, or restricting the treated group to a population with an indication that is not a subclinical stage of the disease) - Excluding a specific period of time prior to the date of diagnosis of the disease (lag-time) from the etiologic window
Losses to follow-up (informative censoring)	- Inclusion of variables that affect censoring and event times in the multivariate regression model - Inverse probability of censoring weighting - Sensitivity analysis
Depletion of susceptibles (prevalent user bias)	- New-user design - Meta-analysis of clinical trials
Missing data	- Replacing each absent observation with a mean value based on observed values of the variable or the predicted value based on a regression model - Imputation methods (e.g. multiple imputation) - Likelihood-based methods - Inverse probability weighting
Measurement bias	
Misclassification bias	- Validation study (exposure/outcome/confounders) + (sensitivity analysis/misclassification control techniques using multivariate regression)
Time-related bias	
Immortal time bias	- Data analysis with procedures that take into account time-dependent exposure in a cohort - Transferring the start of treatment to the end of the immortal time period in both groups
Immeasurable time bias	- Data analysis accounting for the time-varying exposable period
Time-window bias	- Accounting for duration of treatment in the selection of controls - Time-dependent analysis
Time-lag bias	- Comparing patients at the same stage of disease

## Additional file

Additional file 1: Details of articles included in the review. (PDF 227 kb)

## Abbreviations

MeSH: Medical Subject Headings
PRISMA: Preferred Reporting Items for Systematic reviews and Meta-Analyses
