# Supplementary material for: Bias in pharmacoepidemiologic studies using secondary health care databases: a scoping review
Source: BMC Med Res Methodol. 2019 Mar 11;19:53. doi: 10.1186/s12874-019-0695-y (PMC6419460; doi:10.1186/s12874-019-0695-y)
Supplement: Supplementary file 1 — Details of articles included in the review. (PDF 227 kb) [file 12874_2019_695_MOESM1_ESM.pdf]

Additional file 1

Details of articles included in the review

First author [Reference] (Country)	Publication date	Journal category[*]	Type of article	Type of bias[†]			
				Confounding	Selection	Measurement	Time-related
Weinstein RB [80] (USA)	2017 Dec.	PUB	Analysis	CIN, U/RC			
Pottegård A [81] (Denmark)	2017 Dec.	TOX	Review	TDC, U/RC	PRO		
Wade RL [152] (USA)	2017 Sep.	HCS	Analysis			MISE	
Melamed A [82] (USA)	2017 Sep.	OBS	Review	CIN, U/RC			
Czwikla J [153] (Germany)	2017 Aug.	HCS	Analysis			MISO	
Palmaro A [165] (France)	2017 May	PUB	Analysis				ITB
Cepeda MS [140] (USA)	2017 Apr.	PUB	Analysis		MD	MISE	
Dong YH [83] (USA)	2017 Mar.	MGI	Analysis	U/RC	DEP		IMT
Camplain R [141] (USA)	2017 Apr.	PUB	Analysis		MD	MISO	
Bourbeau J [84] (Canada)	2017 Feb.	RES	Review	CIN (confounding by disease severity)	SEL		
Palmaro A [142] (France)	2016 Dec.	PHA	Review		SEL		ITB
Macías D [85] (Spain)	2016 Dec.	PUB	Opinion	CON			
Hudson M [86] (Canada)	2016 Jun.	RHE	Review	CIN (confounding by disease severity), U/RC			IMT
Pauly NJ [154] (USA)	2016 Jun.	HCS	Analysis			MISE	
Mazzali C [155] (Italy)	2016 Jun.	HCS	Review			MISO	
Bérard A [87] (Canada)	2016 Apr.	TOX	Opinion	CIN	MD	MISE, MISO	IMT
Haneuse S [88] (USA)	2016 Apr.	HPS	Review	CON	SEL	MIS	
Filion KB [89] (Canada)	2016 Mar.	PUB	Analysis	CIN, U/RC			
Hampp C [156] (USA)	2016 Mar.	PUB	Analysis			MISE	
Haneuse S [63] (USA)	2016 Jan.	PUB	Analysis		MD		

Golozar A [90] (USA)	2016 Jan.	END	Review	CIN (confounding by disease severity)	DEP		IMT, TWB
Targownik LE [166] (Canada)	2015 Dec.	GAS	Review				IMT
Willis AW [91] (USA)	2015 Nov.	NEU	Review	HUE	SEL	MISE, MISO	
Lanes S [143] (USA)	2015 Oct.	PHA	Review		MD	MISO	
Swanson SA [92] (USA)	2015 Sep.	PHA	Analysis	CIN (confounding by disease severity), TDC, U/RC			
Heinze G [93] (Austria)	2015 Aug.	TRA	Analysis	U/RC	MD	MISE, MISO	
Moore TJ [6] (USA)	2015 Jul.	PUB	Opinion	CIN, U/RC		MISO	
Herrett E [144] (United Kingdom)	2015 Jun.	PUB	Review		MD	MIS	
Moulis G [94] (France)	2015 Jun.	MGI	Review	CON	MD	MISE, MISO	
Jensen ET [95] (USA)	2015 Mar.	PUB	Analysis	CON	SEL		
Patorno E [40] (USA)	2015 Mar.	PUB	Review	CIN, TDC, over-adjustment	DEP, PRO, INC, competing risks	MISE, MISO, temporal ambiguity, detection bias, misclassification of confounders	IMT
Weil G [145] (France)	2015 Feb.	ANE	Review		MD		
Funk MJ [96] (USA)	2014 Dec.	–	Review	CIN, HUE		MISE, MISO, misclassification of confounders	
Patorno E [41] (USA)	2014 Nov.	END	Review	CIN, TDC, over-adjustment	DEP, PRO, INC, competing risks	MISE, MISO, temporal ambiguity, detection bias, misclassification of confounders	IMT
de Groot MC [54] (Netherlands)	2014 Oct.	PUB	Analysis (simulation)		Referral bias	MISE, MISO	
Li X [146] (USA)	2014 Sep.	HPS	Analysis		MD	MISE	
Matok I [167] (Canada)	2014 Sep.	TOX	Reanalysis				IMT
Kim DH [14] (USA)	2014 Sep.	PHA	Review	U/RC			
Winterstein AG [157] (USA)	2014 Aug.	PHA	Analysis			MISO	
Datta R [97] (USA)	2014 Jun.	–	Reanalysis	CIN			
Schneeweiss S [98] (USA)	2014 Jun.	MGI	Opinion	CON			

Yang X [99] (China)	2014 Jun.	–	Opinion	CIN	DEP		IMT
Zhang J [100] (USA)	2014 May	RHE	Review	CIN (confounding by disease severity)			
Gavrielov-Yusim N [101] (Israel)	2014 Mar.	PUB	Review	U/RC	SEL	MISE, MISO	IMT
Cook EA [168] (USA)	2013 Nov.	–	Analysis				ITB
Paxton C [102] (USA)	2013 Nov.	–	Review	CON	SEL		
Gallego B [103] (Australia)	2013 Nov.	HCS	Opinion	U/RC	SEL		
Ryan PB [104] (USA)	2013 Sep.	–	Analysis	CIN, TDC			
Skurtveit S [158] (Norway)	2013 Sep.	HCS	Analysis			MISE	
Hersh WR [105] (USA)	2013 Aug.	HPS	Review	CON	SEL	MIS	TIM
Lauffenburger JC [147] (Canada)	2013 Aug.	PHA			MD	MISE	
Maciejewski ML [148] (USA)	2013 Aug.	HPS	Reanalysis		DEP		
Ali A [57] (USA)	2013 Mar.	–	Review	CIN, TDC	INC, DEP		IMT
Suissa S [106] (Canada)	2012 Dec.	END	Review	CIN			IMT, TWB, TLB
Yang X [107] (China)	2012 Dec.	END	Opinion	CIN	DEP		IMT
Hershman DL [108] (USA)	2012 Dec.	ONC	Review	U/RC	MD	MIS	
Suling M [109] (Germany)	2012 Dec.	–	Review	CON	PRO		
Kiri VA [110] (United Kingdom/ Ireland)	2012 Sep.	PUB	Review	U/RC, CIN (confounding by disease severity)		MISE, MISO	
Yang XL [111] (China)	2012 Jul.	END	Review	CIN,	DEP		IMT
Gamble JM [55] (Canada)	2012 Jun.	HCS	Analysis (simulation)			MISE	
van Walraven C [8] (Canada)	2012 Feb.	PUB	Review			MISO	IMT
Takahashi Y [58] (Japan)	2012 Feb.	PHA	Review	U/RC, TDC	MD	MISE, MISO	
Gamble JM [159] (Canada)	2012 Jan.	PHA	Analysis			MISE	
Nelson JC [112] (USA)	2012 Jan.	PHA	Analysis (simulation)	CON	MD	MISO	
van Walraven C [160] (Canada)	2011 Oct.	PUB	Analysis			MISO	
Fung V [113] (USA)	2011 Jul.	HPS	Review	CIN, U/RC	SEL		

Zhang J [114] (USA)	2011 Jun.	–	Review	CIN		MISO	IMT
Hernan MA [115] (USA)	2011 May	PUB	Opinion	CON	SEL	MIS	
Hoover KW/ Grimes DA [161] (USA) (see #149)	2011 Mar.	OBS	Letter to the editor/ author response			MISO	
Suissa S [169] (Canada)	2011 Mar.	PUB	Reanalysis				TWB
Ray WA [2] (USA)	2011 May	PUB	Opinion	HUE	DEP	MISE, MISO	IMT
Dore DD [162] (USA)	2011 Feb.	PHA	Analysis			MISO	
Grimes DA [149] (USA)	2010 Nov.	OBS	Opinion		SEL	MISO	
McNeil JJ [116] (Australia)	2010 Oct.	–	Opinion	CIN, U/RC	INC, DEP, MD		
Ehrenstein V [117] (Denmark)	2010 Aug.	–	Review	CON	SEL	MEA	
Hudson M [118] (Canada)	2010 Jun.	RHE	Review	CIN (confounding by disease severity)	DEP		IMT
Brookhart MA [18] (USA)	2010 Jun.	HPS	Review	CIN (confounding by disease severity), HUE, U/RC (confounding by frailty), bias resulting from statistical control of an intermediate variable, instrumental variable (Z-bias) or collider-stratification bias (M-bias)	Health-care differential access bias		
Velthove KJ [150] (Netherlands)	2010 May	–	Analysis		Testing bias, diagnostic suspicion bias, referral bias		
Martin–Latry K [119] (France)	2010 Mar.	PHA	Review	U/RC	MD	MISE	
Lévesque LE [68] (Canada)	2010 Mar.	–	Reanalysis				IMT
Giezen TJ [120] (Netherlands)	2009 Oct.	PUB	Review	CIN (confounding by disease severity)			
Kiri VA [77] (United Kingdom/ Ireland) (see #69)	2009 Sep.	PUB	Letter to the editor				IMT
Harpe SE [121] (USA)	2009 Feb.	PHA	Review	CON	SEL	MISE, MISO	
Schneeweiss S [15] (USA)	2009 Feb.	NEU	Analysis	U/RC (confounding by frailty)			
Suissa S [170] (Canada)	2008 Aug.	PUB	Reanalysis				ITB

Giordano SH [122] (USA)	2008 Jun.	–	Analysis	CIN	SEL		
Pigeot I [123] (Germany)	2008 Mar.	PHA	Review	CON			
Suissa S [69] (Canada)	2008 Feb.	PUB	Review				IMT
Strom BL [16] (USA)	2007 Oct.	HPS	Review	CON	SEL	MISE, MISO	
Cramer JA [124] (USA)	2007 Oct.	MGI	Analysis	CON	SEL	MEA	
Terris DD [125] (USA)	2007 Jul.	PUB	Review	HUE	MD	MISO	
Hughes MD [126] (USA)	2007 Apr.	MGI	Opinion	CIN			
Suissa S [70] (Canada)	2007 Mar.	PHA	Review				IMT
Lanes SF [163] (USA)	2006 Nov.	TOX	Analysis			MISO	
Smeeth L [22] (United Kingdom)	2006 Oct.	PUB	Opinion	CIN	SEL		
De Vries F [127] (Netherlands)	2006 Oct.	PUB	Reanalysis	U/RC, HUE			
Suissa S/ Kiri VA [128] (Canada/ United Kingdom)	2006 Feb.	RES	Letter to the editor/ author response	CIN (confounding by disease severity)			IMT
Hennessy S [1] (USA)	2006 Mar.	TOX	Review	U/RC			
Etminan M [129] (Canada)	2006 Jan.	PHA	Review	CIN			IMT
Ray WA [130] (USA)	2005 Dec.	MGI	Opinion	CIN, U/RC		MISE	
Holbrook A [131] (USA)	2005 Oct.	–	Review	CIN			
Schneeweiss S [62] (USA)	2005 Jun.	GER	Analysis	U/RC			
Suissa S [71] (Canada)	2005 Apr.	ALL	Reanalysis				IMT
Schneeweiss S [3] (USA)	2005 Apr.	PUB	Review	CIN, U/RC		MISE, MISO	
Schneeweiss S [132] (USA)	2004 Dec.	PSY	Analysis	U/RC			
Kiri VA/ Suissa S [133] (United Kingdom/ Canada) (See #72)	2004 Jul.	RES	Letter to the editor/ author response	CIN			IMT
Suissa S [72] (Canada)	2004 Jul.	RES	Reanalysis				IMT
Bannwarth B [134] (France)	2004 Jul.	–	Opinion	CIN, U/RC			
Zhan C [135] (USA)	2003 Dec.	–	Review	CON	MD	MISO	

Sin DD [73]/ Pride NB [74] (Canada) (See #75)	2003 Jul.	RES	Letter to the editor					IMT
Suissa S [75] (Canada)	2003 Jul.	RES	Reanalysis					IMT
Oostenbrink R [151] (Netherlands)	2003 Jun.	PUB	Opinion			MD	Verification/ referral/workup bias, incorporation/test review/diagnostic review bias	
Ray WA [164] (USA)	2002 Dic.	PHA	Analysis				MISE	
Strom BL [136] (USA)	2001 Aug.	PHA	Review	U/RC		MD	MISO	
Sørensen HT [137] (Denmark)	2001 Jun.	–	Review	CON		MD	MISO	
Hallas J [138] (Denmark)	2001 Jun.	–	Review	CIN			MISE	
Skegg DC [139] (New Zealand)	2000 Nov.	–	Opinion	U/RC				

[*] Journal category in the year of publication of the article according to the Journal Citation Reports®, available at https://jcr.incites.thomsonreuters.com/. If the journal was indexed under more than one category, the category under which it was best ranked was considered. Abbreviations: ALL, Allergy; ANE, Anesthesiology; END, Endocrinology & metabolism; GAS, Gastroenterology & hepatology; GER, Geriatrics & gerontology; HCS, Health care sciences & services; HPS, Health policy & services; MGI, Medicine, general & internal; NEU, Clinical neurology; OBS, Obstetrics & gynecology; ONC, Oncology; PHA, Pharmacology & pharmacy; PSY, Psychiatry; PUB, Public, environmental & occupational health; RES, Respiratory system; RHE, Rheumatology; TOX, Toxicology; TRA, Transplantation; –, Not indexed.

[†] The categories/subcategories of biases are described in Table 1. If the article mentions a subcategory of bias different to those indicated in Table 1, the name of the bias is specified. If the article does not mention a subcategory of bias, the acronym for the general category is used: CON, Confounding; CIN, Confounding by indication; TDC, Time-dependent confounding; U/RC, Unmeasured/residual confounding; HUE, Healthy user/adherer effect; SEL, Selection bias; PRO, Protopathic bias; INC, Losses to follow-up (informative censoring); DEP, Depletion of susceptibles (prevalent user bias); MD, Missing data; MEA, Measurement bias; MIS, Misclassification bias; MISE, Misclassification of exposure; MISO, Misclassification of outcome; TIM, Time-related bias; IMT, Immortal time bias; ITB, Immeasurable time bias; TWB, Time-window bias; TLB, Time-lag bias.
